# Work Hours and Difficulty in Leaving Work on Time in Relation to Work-to-Family Conflict and Burnout Among Female Workers in Taiwan

**DOI:** 10.3390/ijerph17020605

**Published:** 2020-01-17

**Authors:** Shu-Ling Huang, Ren-Hau Li, Shu-Yi Fang, Feng-Cheng Tang

**Affiliations:** 1 Department of Psychology, Chung Shan Medical University, Taichung 402, Taiwan; shuling@csmu.edu.tw (S.-L.H.); davidrhlee@yahoo.com.tw (R.-H.L.); 2 Room of Clinical Psychology, Chung Shan Medical University Hospital, Taichung 402, Taiwan; elice0625@gmail.com; 3 Department of Leisure Services Management, Chaoyang University of Technology, Taichung 413, Taiwan; 4 Department of Occupational Medicine, Changhua Christian Hospital, Changhua 500, Taiwan; 5 School of Medicine, Chung Shan Medical University, Taichung 402, Taiwan

**Keywords:** work hours, work-to-family conflict, burnout, leaving work on time, women

## Abstract

The present study explores the relations between work hours and the difficulty in leaving work on time to both work-to-family conflict (WFC) and burnout among female workers in Taiwan. A cross-sectional research design and questionnaire were employed to obtain the research data. In total, 738 full-time female workers took part in the study. The results of regression analyses showed that when age, marital status, economic status, occupation, parental status, and housework responsibilities were controlled, more work hours were positively associated with WFC and burnout. When the difficulty in leaving work on time was also considered in the analysis, long working hours were still significantly associated with burnout; however, the significant relation with WFC disappeared. It is surmised that if female employees work overtime voluntarily, the perception of WFC diminishes; nevertheless, the adverse effect of long working hours on health remains unabated. This study concludes that female employees who work overtime on a voluntary basis are at risk of health problems, which should be a focus of concern.

## 1. Introduction

Due to the increasing influx of female workers in Taiwan, concern has arisen with regard to the work-life balance of women, who traditionally have had more domestic responsibilities than men. With their growing participation in the labor force, the demands on women are becoming greater. In 1978, the female labor force participation rate in Taiwan was 39.1%, whereas in 1988 it was 45.6%. The latest data for 2019 showed it was 51.5% [1,2]. However, female workers were more vulnerable to physical and psychological symptoms than male workers in Taiwan [3]. Another survey for hospital staff in Taiwan also showed that when job position, work year, and daily work time were taken into consideration, female employees experienced more nervousness, nightmares, irritability, headaches, and insomnia than male employees did [4]. Studies have shown that female workers are generally more vulnerable to occupational stress than are male workers [5,6]. One study reported that stress levels of female managers were as high after work as during work, whereas among male managers, stress levels rapidly decreased when the working day ended [7]. Even when physical inactivity was controlled for in the analysis, gender differences existed in the presentation of burnout [8]. Exhaustion was consistently found to be more prevalent among women than men [9].

Work-to-family conflict (WFC) has shown to be a longitudinal predictor of positive wellbeing among employees, even when controlling for social desirability bias [10]. Previous research has also documented work-related, non-work-related and stress-related outcomes [11]. Of particular interest to this study is the finding that WFC is more strongly associated with exhaustion in women than in men [9]. The value of these studies cannot be questioned; however, previous researches usually assume that WFC is an operative construct independent of cultural context. Since the term was coined in the West, a full picture of WFC in the Asian context requires an understanding of Eastern traditions and values. A previous study in Hong Kong found a non-significant relation between WFC and strain that was inconsistent with most findings in the West [12]. Another cross-cultural study found that workload was significantly associated with WFC among British study subjects, but not among Taiwanese subjects [13]. More studies in the East are needed to gain a fuller understanding of WFC. 

Burnout is an important issue in psychological literature. The term burnout is defined as a work-related mental health impairment comprising three dimensions: emotional exhaustion, cynicism, and inefficacy [14]. Burnout can result in adverse physical, psychological and occupational consequences. A systematic review of these consequences found that burnout is significantly related to physical health problems, such as type 2 diabetes, coronary heart disease, musculoskeletal pain, headaches, gastrointestinal issues, and so on; psychological effects include insomnia and depressive symptoms, among others; occupational outcomes extend from job dissatisfaction to absenteeism and new disability pension [15]. In addition, burnout was especially prevalent in certain occupations and among certain practitioners, for example physicians, of whom 46% had at least one symptom of burnout, according to a study in the USA [16]. Approximately one-third of physicians in the UK exhibited aspects of burnout [17]. Among the general population, a Finnish nationwide health survey found that 27.6% of general employees suffered from mild or severe burnout [18]. Due to the overtly adverse outcomes of burnout documented in these studies, which cover different areas of the world, this issue cannot be overlooked in studies on health and wellbeing among the labor force in Asia. An early identification of burnout may benefit employee health and reduce national health costs.

Among European Union workers, high satisfaction with working hours has been associated with high satisfaction with work-life balance [19]. Since 2017, the Taiwan government has tried to decrease work hours by amending the Labor Standards Act; however, the effects are still too recent to be reflected in the literature [20]. According to data from the 2018 Organization for Economic Cooperation and Development (OECD) and the Ministry of Labor in Taiwan, the average annual working hours in Taiwan are 2033. This figure exceeds those in Korea, Japan, and most countries in the OECD [21]. As with burnout, harmful effects of long working hours on health have been reported. One study found a substantial correlation between the number of working hours per week and the frequencies of both musculoskeletal disorders and psycho-vegetative complaints [22]. Several studies have suggested a potential association of exposure to long working hours with increased risks of cardiovascular diseases in general, and coronary heart disease and stroke specifically [23]. A systematic review of epidemiological evidence revealed that long working hours are associated with a depressive state, anxiety, and sleep disorders [24]. Strategies that might be applied to reduce the working hours in Taiwan have not yet been fully considered within occupational health care.

Working long hours does not necessarily equal being unable to leave work on time. While the latter has consistently been regarded as a complaint pertaining to work strain, its effect on WFC or burnout has seldom been examined [25,26,27]. Most employees do not welcome compulsorily overtime; a more flexible work schedule and higher level of autonomy with respect to time management might alleviate complaints. Accordingly, some governments and employers have adopted friendly working arrangement policies, such as flexible work hours, longer lunch breaks and regulations against compulsory overtime [28]. To conclude that these initiatives result in an automatic reduction of working hours, however, would be erroneous. The work conditions are now fast-changing. “Work is a big part of our lives, but it’s now easier than ever to let it take over. Mobile devices that are supposed to free us from the office can actually bind us to it” [29]. The boundary between work and life is becoming vague. The association between working long hours on a voluntary basis and employee perceptions is not yet clear. Therefore, both work hours and the difficulty in leaving work on time were considered simultaneously in the present study with the aim of exploring their effects on WFC and burnout.

## 2. Materials and Methods

### 2.1. The Participants and Recruitment Method

The present study was carried out from 2013 to 2014 by the Center for Workplace Health Promotion, using a cross-sectional research method with convenience sampling. This study was conducted according to the Declaration of Helsinki and was approved by the Institutional Review Board of the Changhua Christian Hospital in Taiwan with a waiver of informed consent. Full-time female workers 20 years of age or older were recruited from six manufacturing companies in Central Taiwan to participate in this study. A consent form was distributed with the survey questionnaire. In total, 738 full-time Taiwanese female employees agreed to voluntarily take part in the study. A more detailed recruitment procedure is described in a previous paper [30]. 

### 2.2. Measures

The survey questionnaire comprised several sections: personal information, family- and work-related questions, WFC, and burnout. Personal information included age, marital status, occupation, and economic status. Occupation was divided into two categories: white-collar (including management, professional, technician, office worker, and service worker) and blue-collar (including crafts worker and machine operator) [31]. Using a five-point Likert response format ranging from 1 “very poor” to 5 “excellent,” the study measured economic status with one question: “How would you rate your economic status at the present time?” 

Family-related questions included the burden of parenting and the responsibility of housework. “Do you look after any child/grandchild under 12 years old at home?” was asked with a Yes or No response option. “How much housework do you take on?” was asked with a five-point Likert response format ranging from 1 “almost none” to 5 “almost all.” Work-related questions included working hours per week and the difficulty in leaving work on time. Using a four-point Likert response format ranging from 1 “not difficult at all” to 4 “very difficult,” the study measured the difficulty in leaving work on time with one question: “In general, how would you rate the difficulty in getting off work on time during the recent month?” 

The level of work-to-family conflict (WFC) was measured using five questions adopted from the work–home interference scale [32]. This questionnaire was chosen for the present study because the items were not limited to married individuals. This scale assessed both time-based and strain-based conflict, and exhibited good internal reliability, with Cronbach’s alpha equaling 0.820. This questionnaire used a four-point scale ranging from 1 (never) to 4 (always). Higher scores indicated higher levels of WFC. The Cronbach’s alpha was 0.703 in the present study.

The Copenhagen Burnout Inventory (CBI) was adopted to assess the level of burnout among respondents [33]. The CBI is a valid and reliable questionnaire measuring three burnout sub-dimensions: personal burnout (6 items), work-related burnout (7 items), and client-related burnout (6 items) [33,34]. According to the theoretical rationale of the CBI, the three subscales can be used independently in accordance with the populations being studied [33]. For the purposes of the present study, two subscales of personal and work-related burnout were adopted to form a mean as general burnout. In this study, Cronbach’s alpha was 0.940 for general burnout. Responses were made on a 5-point scale ranging from 0 (never) to 4 (always). The scale labels were then re-coded into 0 (never), 25, 50, 75, and 100 (always). Higher scores indicated more burnout. 

### 2.3. Statistical Analysis

The demographic characteristics of the participants, along with the surveyed variables, were summarized using descriptive statistics. The chi-square test or *t*-test was used to compare the differences by occupation. Pearson’s correlation was used to analyze the relationships between the continuous variables. Multiple linear regression was used to investigate the relations among work hours, the difficulty in leaving work on time, WFC, and burnout. Among the controlled factors, unmarried workers and white-collar workers were coded as 0; married workers and blue-collar workers were coded as 1. All statistical procedures were performed using SPSS statistics 20 (SPSS Inc., Chicago, IL, USA); a *p*-value less than 0.05 was considered statistically significant. 

## 3. Results

To ensure the credibility of the results, we excluded questionnaires with more than 20% of the question items incomplete, leaving a total of 738 questionnaires successfully collected. Table 1 lists the demographic characteristics, WFC, burnout, and other research variables of the participants by occupation. Of the participants, the majority were aged 40–49 (35.9%) or 50–59 (35.4%), married (76.7%), and white-collar workers (81.3%). About two-thirds of the participants perceived their economic status as ordinary (63.4%); 77.7% of the participants did not look after any child at home. The comparisons of age, economic status, parental status, work hours, WFC, and burnout by occupation showed significant differences. White-collar employees showed fewer work hours and a lower level of burnout compared to blue-collar employees, but a higher level of WFC.

The crude correlations of research variables are listed in Table 2. The responsibility of housework was significantly negatively correlated with work hours and the difficulty in leaving work on time, but not with WFC or burnout. Work hours, the difficulty in leaving work on time, WFC, and burnout were all significantly positively associated with one another.

Multiple linear regressions predicting WFC are summarized in Table 3. In Model 1, with demographic factors and family-related factors controlled, the model for work hours predicting WFC was significant (F = 9.900, *p* < 0.001; R^2^ = 0.088); work hours showed a significantly positive relation with WFC (β = 0.247, *p* < 0.001). In Model 2 (F = 21.526, *p* < 0.001; R^2^ = 0.194), with the difficulty in leaving work on time also considered and controlled for in the analysis, the significant effect of work hours on WFC disappeared (β = 0.073, *p* = 0.082). However, the difficulty in leaving work on time showed a significant association with WFC (β = 0.373, *p* < 0.001).

Multiple linear regressions predicting burnout are also summarized in Table 4. In Model 1, with demographic factors and family-related factors controlled, the model for work hours predicting burnout was significant (F = 24.321, *p* < 0.001; R^2^ = 0.192); work hours showed a significantly positive relation with burnout (β = 0.251, *p* < 0.001). In Model 2 (F = 25.853, *p* < 0.001; R^2^ = 0.224), with the difficulty in leaving work on time also considered and controlled for in the analysis, work hours remained positively associated with burnout (β = 0.155, *p* < 0.001). In addition, the difficulty in leaving work on time also showed a significantly positive association with burnout (β = 0.206, *p* < 0.001).

## 4. Discussion

The main purpose of this study was to evaluate the relations of work hours and the difficulty in leaving work on time to both WFC and burnout among female workers in Taiwan. The results showed that in the multiple linear regression analyses, when age, marital status, economic status, occupation, parental status, and the responsibility of housework were all controlled, a higher number of work hours was positively associated with WFC and burnout. When the difficulty in leaving work on time was also considered and controlled for in the analysis, work hours remained significantly positively associated with burnout; however, the significant effect on WFC disappeared.

The adverse effects of long working hours on physical and mental health have been evidenced [22,23,24]. A high number of work hours was positively associated with burnout in the present study. This finding is in line with another study conducted in Taiwan; a comparison of high- and low-burnout groups found that long working hours was significantly correlated with burnout in a dose-dependent manner even when physical inactivity was controlled [8]. However, in the present study, the significant association between work hours and WFC disappeared when the difficulty in leaving work on time was considered. Being unable to leave work on time has generally been recognized as a sign of job strain [25]. In modern times, however, many workers have the ability to control their work schedules. When the hazardous effects of long working hours on health are gradual and unobvious, and feelings of WFC decline, workers may choose to spend more time at work unaware of the potential harm. WFC was broadly evidenced to be associated with burnout, depression, and psychological strain [11]. These voluntary extended hours should be a matter of concern.

Notably, economic status showed a significant association with both WFC and burnout when the burden of parenting, the responsibility of housework, occupation, and work hours were all considered. Previous research has indicated associations between socioeconomic status and both physical and mental health. A sixteen-year longitudinal study revealed that a change in occupation and income were both related to changes in health. A causal relationship between these variables indicates that initial socioeconomic status affects later changes in morbidity [35]. In addition, a study of 29 countries using European social surveys showed that individual economic conditions were a basic factor contributing to a good state of self-reported health [36]. Regarding mental health, common mental disorders were found to be distributed according to a gradient of economic disadvantage across society [37]. Finally, a systematic review of epidemiological literature found that of the 115 studies reviewed, over 70% reported positive associations between poverty and common mental disorders in low and middle-income countries [38]. Despite these findings, economic status has seldom been discussed in the field of occupational health; this is an obvious oversight. To promote mental health in the workplace, occupational health officials should be concerned about, and further investigate, workers’ economic status.

The perception of WFC reflects not only time- and strain-based conflict, but also an awareness of the importance of achieving a balance between work life and family life [11]. In the present study, blue-collar female employees reported having significantly longer working hours and higher levels of burnout, along with significantly lower levels of WFC, compared to white-collar female employees. The lower WFC among blue-collar female employees might imply more willingness to put family matters second to work and/or a resignation to destiny. Traditionally in Taiwanese culture, being a “good wife and wise mother” has represented the ideal for womanhood, and is more important than realizing one’s personal identity [39]. However, in the modern world, women face previously unknown dilemmas, which can lead to an attempt to shake off the constraints posed by traditional male rights, in asserting their own place in society and affirming their unique existence [40]. Accordingly, more and more women have begun to enroll in higher education and to devote themselves to the labor force [40]. As stated in the Introduction, the female labor force participation rate in Taiwan has increased from about one third in the 1970s to more than one half in the current year [1]. Considering these changes taking place, more research needs to be conducted that addresses the impact of cultural tradition on female identity and self-perception, particularly as it relates to WFC. Further research is also needed to clarify whether the phenomenon in the present study, that is, blue-collar female employees working comparatively more at home and on the job but feeling less WFC, is rooted in the Taiwanese culture or whether other factors pertaining to socioeconomic status are involved.

There were some limitations to this study. First, the cross-sectional study design did not allow for the determination of the developmental process and the causal relationships among the research variables. More longitudinal studies are required to explain the mechanism governing the relations among the research variables. Second, the participants took part voluntarily in the present study; thus, self-selection bias could not be ruled out. In addition, all the research variables were obtained via subjective data. Therefore, applying the research results to other populations should be done with caution. Third, the number of blue-collar female employees was small (19%) in the present study. At the time of this study, blue-collar female employees in Taiwan occupied about 31% of the labor force [1]. Enlarging the sample of blue-collar employees in future research is recommended.

## 5. Conclusions

The present study explored the relations of work hours and the difficulty in leaving work on time to both work-to-family conflict (WFC) and burnout among female workers. In the regression analyses, when age, marital status, economic status, occupation, parental status, and housework were all controlled, a high number of work hours was positively associated with WFC and burnout. When the difficulty in leaving work on time was also controlled for in the analysis, work hours was still significantly associated with burnout; however, the significant association between work hours and WFC disappeared. Although the adverse effect of long working hours on health has been evidenced, its hazardous effects are gradual and unobvious. Additionally, when feelings of WFC decline, workers may choose to spend more time at work, oblivious to the potential harm they are causing themselves. The deleterious effects of extended hours on employee health should be a concern. In addition, the present study showed that economic status is significantly associated with both WFC and burnout. Nevertheless, the effects of economic status on employees’ mental health are seldom examined in the field of occupational health. Further studies regarding this issue are recommended.

## Figures and Tables

**Table 1:** Comparing demographic characteristics and research variables by occupation.

Variables	Total (*n* = 738)	White-Collar (*n* = 600)	Blue-Collar (*n* = 138)		
	*n* (%) ^a^	*n* (%) ^a^	*n* (%) ^a^	χ^2^	
Age (year)				30.494	***
20–29	51 (6.9)	41 (6.8)	10 (7.2)		
30–39	143 (19.4)	97 (16.2)	46 (33.3)		
40–49	265 (35.9)	213 (35.5)	52 (37.7)		
50–59	261 (35.4)	235 (39.2)	26 (18.8)		
60 and over	18 (2.4)	14 (2.3)	4 (2.9)		
Marital status				0.499	
not married	172 (23.3)	143 (23.8)	29 (21.0)		
married	566 (76.7)	457 (76.2)	109 (79.0)		
Economic status					
very poor	39 (5.3)	23 (3.9)	16 (11.6)	33.007	***
poor	11 (1.5)	7(1.2)	4 (2.9)		
ordinary	464 (63.4)	365 (61.4)	99 (71.7)		
good	190 (26.0)	176 (29.6)	14 (10.1)		
excellent	28 (3.8)	23 (3.9)	5 (3.6)		
Parental status				30.037	***
no	570 (77.7)	487 (81.7)	83 (60.1)		
yes	164 (22.3)	109 (18.3)	55 (39.9)		
Variables (possible range)	Mean (SD)	Mean (SD)	Mean (SD)	*t*	
Housework (1–5)	3.29 (1.02)	3.26 (1.00)	3.43 (1.10)	−1.765	
Work hours (27–72)	43.51 (7.05)	42.19 (5.11)	49.21 (10.63)	−7.556	***
Difficulty in leaving work on time (1–4)	1.52 (0.79)	1.53 (0.76)	1.46 (0.87)	0.991	
WFC ^b^ (5–20)	9.09 (2.13)	9.26 (2.05)	8.39 (2.35)	4.347	***
Burnout (0–52)	20.30 (8.60)	19.78 (8.05)	22.56 (10.39)	−2.951	**

^a^ Calculated according to a percentage of the valid count. ^b^ WFC denotes work-to-family conflict. **p < 0.01; *** *p* < 0.001 calculated using *t* or χ^2^ test.

**Table 2:** The correlations of research variables (*n* = 738).

Variables	Housework	Work Hours	Difficulty in Leaving Work on Time	WFC
Housework	-			
Work hours	−0.093 *	-		
Difficulty in leaving work on time	−0.140 **	0.422 **	-	
WFC	0.025	0.151 **	0.400 **	-
Burnout	0.043	0.303 **	0.306 **	0.326 **

* *p* < 0.05; ** *p* < 0.01.

**Table 3:** Multiple regression for WFC.

Variables	Model 1				Model 2			
	B	SE B	*β*	*p*	B	SE B	*β*	*p*
Constant	6.127	0.759			6.186	0.714		
Age	0.084	0.096	0.038	0.381	0.150	0.090	0.068	0.098
Marriage	0.065	0.199	0.013	0.743	0.108	0.187	0.021	0.566
Economic status	−0.222	0.084	−0.100	0.009	−0.179	0.079	−0.081	0.024
Occupation	−1.520	0.216	−0.280	<0.001	−1.026	0.209	−0.189	<0.001
Parental status	0.224	0.204	0.044	0.272	0.136	0.192	0.027	0.478
Housework	0.081	0.080	0.039	0.313	0.133	0.076	0.064	0.080
Work hours	0.074	0.012	0.247	<0.001	0.022	0.013	0.073	0.082
Difficulty in leaving work on time	--	--	--	--	1.014	0.105	0.373	<0.001
R^2^	0.088				0.194			
∆R^2^					0.106			
F	9.900 ***				21.526 ***			

Β denotes unstandardized regression coefficient; SE B denotes standard error; *β* denotes standardized regression coefficient. *** *p* < 0.001.

**Table 4:** Multiple regression for burnout.

Variables	Model 1				Model 2			
	B	SE B	*β*	*p*	B	SE B	*β*	*p*
Constant	16.887	2.896			17.018	2.839		
Age	−1.606	0.366	−0.179	<0.001	−1.458	0.360	−0.162	<0.001
Marriage	0.052	0.759	0.003	0.945	0.147	0.745	0.007	0.843
Economic status	−1.823	0.321	−0.204	<0.001	−1.727	0.315	−0.193	<0.001
Occupation	−1.151	0.824	−0.052	0.163	−0.044	0.833	−0.002	0.958
Parental status	0.844	0.779	0.041	0.279	0.647	0.764	0.031	0.398
Housework	0.712	0.307	0.084	0.021	0.827	0.301	0.098	0.006
Work hours	0.306	0.046	0.251	<0.001	0.189	0.050	0.155	<0.001
Difficulty in leaving work on time	--	--	--	--	2.269	0.416	0.206	<0.001
R^2^	0.192				0.224			
∆R^2^					0.032			
F	24.321 ***				25.853 ***			

Β denotes unstandardized regression coefficient; SE B denotes standard error; *β* denotes standardized regression coefficient. *** *p* < 0.001.
